# Small Interfering RNAs Targeting VP4, VP3, 2B, or 3A Coding Regions of Enterovirus A71 Inhibit Viral Replication In Vitro

**DOI:** 10.3390/biomedicines13071760

**Published:** 2025-07-18

**Authors:** Yun Ji Ga, Yun Young Go, Jung-Yong Yeh

**Affiliations:** 1 Department of Life Sciences, College of Life Sciences and Bioengineering, Incheon National University, BioComplex, Harmony-ro 265, Yeonsu-gu, Incheon 22014, Republic of Korea; dkfmal92@inu.ac.kr; 2 College of Veterinary Medicine, Konkuk University, Neungdong-ro 120, Gwangjin-gu, Seoul 05029, Republic of Korea; yunygo@konkuk.ac.kr; 3 Center for Brain-Machine Interface, Incheon National University, BioComplex, Harmony-ro 265, Yeonsu-gu, Incheon 22014, Republic of Korea; 4 Convergence Research Center for Insect Vectors, Incheon National University, BioComplex, Harmony-ro 265, Yeonsu-gu, Incheon 22014, Republic of Korea

**Keywords:** antiviral, enterovirus, RNA interference, small interfering RNA, virus

## Abstract

**Background**: Enterovirus A71 (EV-A71) is considered as the primary causative agent of hand, foot, and mouth disease (HFMD) in young children, leading to severe neurological complications and contributing to substantial mortalities in recent HFMD outbreaks across Asia. Despite this, there is currently no effective antiviral treatment available for EV-A71. RNA interference (RNAi) is a powerful mechanism of post-transcriptional gene regulation that utilizes small interfering RNA (siRNA) to target and degrade specific RNA sequences. **Objectives:** The aim of this study was to design various siRNAs targeting EV-A71 genomic regions and evaluate the RNAi efficacy against a novel, previously genetically uncharacterized EV-A71 strain. **Methods**: A novel EV-A71 strain was first sequenced to design target-specific siRNAs. The viral titers, viral protein expression, cytopathic effects, and cell viability of EV-A71-infected HeLa cells were examined to evaluate the specific viral inhibition by the siRNAs. **Results**: A substantial reduction in viral titers and viral protein synthesis was observed in EV-A71-infected HeLa cells treated with specific siRNAs targeting the VP4, VP3, 2B, and 3A genes. siRNAs delayed cytopathic effects and increased cell viability of EV-A71-infected HeLa cells. Nonspecific interferon induction caused by siRNAs was not observed in this study. In contrast, replication of coxsackievirus B3, another important member of the *Enterovirus* genus, remained unaffected. **Conclusions**: Overall, the findings demonstrate that RNAi targeting genomic regions of EV-A71 VP4, VP3, 2B, or 3A could become a potential strategy for controlling EV-A71 infection, and this promising result can be integrated into future anti-EV-A71 therapy developments.

## 1. Introduction

EV-A71 belongs to the species Human enterovirus A of the genus *Enterovirus* within the family Picornaviridae and is a nonenveloped, (+) polarity single-stranded RNA virus. Its genome is approximately 7.4 kb in length and encodes four structural proteins (VP1-VP4) and seven nonstructural proteins (2A, 2B, 2C, 3A, 3B, 3C, and 3D).

Enterovirus A71 (EV-A71) was first identified in California, USA, in 1969, and since then, cases of hand, foot, and mouth disease resulting from EV-A71 infection have exhibited a prevalent pattern worldwide, with particularly high incidence rates reported in the Asia-Pacific region [1,2,3,4]. Since the successful control of poliovirus, EV-A71 has considered as the most crucial neurotropic enterovirus, and globally, EV-A71 has been responsible for substantial morbidity and mortality. Notably, EV-A71 has recently re-emerged as a severe disease in children [5], and there is an urgent need to develop effective antiviral treatments for EV-A71 infection.

The specific and manageable potential of antiviral siRNAs as gene-translation-inhibitory molecules has been extensively studied over the last decade. Clinical trials are underway for several viral pathogens, including Ebola virus (TKM-Ebola, phase I) [6,7], human immunodeficiency virus (pHIV7-shI-TAR-CCR5RZ, phase I) [8], hepatitis B virus (NucB1000, phase I) [9], hepatitis C virus (SPC3649, phase II) [10], and respiratory syncytial virus (ALN-RSV01, phase II) [11,12]. To date, it has been reported that various RNA interference (RNAi) systems can effectively suppress the proliferation of viruses by targeting several EV-A71 proteins in vitro [13,14,15,16,17,18] and in vivo [19]. In both cell culture and animal studies, RNA interference (RNAi) using chemically synthesized siRNA or plasmid-derived shRNA has successfully inhibited EV71 infection. This approach has identified several potential target sequences within key regions of the EV-A71 genome, including 5′ UTR, VP2, VP1, 2A, 2C, 3B, 3C, 3D, and 3′ UTR. Nevertheless, the presence of unconfirmed EV-A71 proteins as targets of small interfering RNA (siRNA) leads to a deficiency of experimentally validated viral siRNA databases. In addition, compared to the findings on other enteroviruses, such as polioviruses and coxsackieviruses, the impact of siRNA inhibition on several genes with potential as targets for EV-A71 remains unresolved.

In this study, we conducted experiments to assess the effectiveness of siRNA therapies against a previously genetically uncharacterized strain of EV-A71.

## 2. Materials and Methods

### 2.1. Cell Cultures and Viruses

A novel genetically uncharacterized EV-A71 isolate (KBPV-VR-56) was used to test the efficacy of the siRNAs designed in the present study. Viruses were propagated in HeLa cells (CCL-2™, American Type Culture Collection (ATCC), Rockville, MD, USA) cultivated in Dulbecco’s modified Eagle’s medium (DMEM, Invitrogen Life Technologies, Carlsbad, CA, USA) with 10% fetal bovine serum (FBS, Merck Millipore, Darmstadt, Germany) and a 100 μg/mL penicillin/streptomycin cocktail (Invitrogen Life Technologies) in an atmosphere containing 5% CO_2_. In addition, the KR-2002 strain (GenBank accession No. OQ919474) of coxsackievirus B3 (CVB3, National Culture Collection for Pathogens, Osong-eup, Republic of Korea) was used to compare the inhibitory potential of siRNAs in a virus belonging to a different genus, *Enterovirus*.

### 2.2. RT–PCR and Viral Genome Sequencing

To identify the novel EV-A71 strain and rapidly design efficient siRNAs targeting a virus with unknown genomic sequences, we first performed PCR and sequencing of the EV-A71 isolate to be targeted. Total viral RNAs were extracted with a QIAamp Viral RNA Kit (Qiagen, Hilden, Germany) and subjected to reverse transcription using an iScript cDNA Synthesis Kit (Bio-Rad Laboratories, Hercules, CA, USA) according to the manufacturer’s instructions. PCR was performed using AccuPower HotStart Pfu PCR PreMix (Bioneer, Daejeon, Republic of Korea) following the manufacturer’s instructions. RT–PCR and sequencing of the target gene were carried out using a diagnostic primer [20] and primers based on conserved sequences between EV-A71 genotype A. The primer sequences for amplifying the region including the 5′ untranslated regions (UTRs)*,* VP4, VP3, 2B, 3A, and 3B genes of EV-A71 are listed in Table S1.

Whole-genome sequences of EV-A71 were retrieved from the GenBank NCBI database (http://www.ncbi.nlm.nih.gov, accessed on 30 June 2023), and the 5′ UTR of the EV-A71 strain investigated in the present study was identified with complete homology to the BrCr strain using NCBI’s BLAST (Table S1). The BrCr strain, an early prototype, is known to be the only member of EV-A71 genotype A. Based on the BrCr strain, of which the 5′ UTR was confirmed to have complete homology with the EV-A71 strain targeted in this study, primers with sequences conserved within EV-A71 genotype A were constructed, and the sequences of five genes (VP4, VP3, 2B, 3A, and 3B) were confirmed through sequencing (Table S2).

For genomic sequencing, purified PCR products were submitted to Cosmogenetech, Inc. (Seoul, Republic of Korea). Sequence similarity between all available EV-A71 strains worldwide in the GenBank gene sequence database of the National Institutes of Health was assessed using Basic Local Alignment Search Tool (BLAST, https://blast.ncbi.nlm.nih.gov/Blast.cgi, accessed on 30 June 2023) searches for each segment based on the sequencing results.

### 2.3. siRNA Design

**Figure 1:**
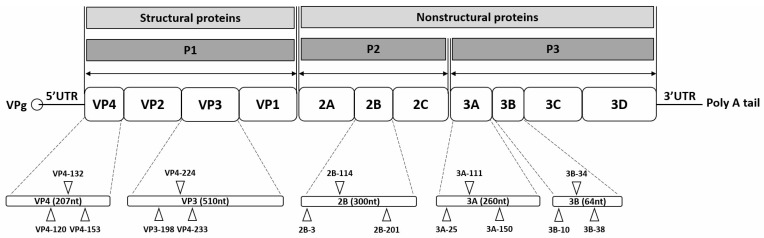
Schematic representation of the enterovirus A71 (EV-A71) genome and siRNA target sites. The EV-A71 RNA genome consists of a single open reading frame with untranslated regions (UTRs) at the 5′ and 3′ ends. At the 5′ UTR end, the genome is covalently connected to viral protein genome-linked (VPg), a small peptide that plays a crucial role in protein priming and replication. The 3′ UTR incorporates a poly-A tail. The open reading frame encodes a polyprotein that includes four structural proteins (VP4, VP2, VP3, and VP1) in the P1 region, as well as seven nonstructural proteins (2A, 2B, 2C, 3A, 3B, 3C, and 3D) in the P2 and P3 regions. Fifteen target sites are indicated on the EV-A71 genome, corresponding to specific siRNAs listed in Table 1. Each target site is labeled with the name of the corresponding siRNA.

Based on the genetic information obtained from the sequence analysis described above, double-stranded siRNAs targeting the VP4, VP3, 2B, 3A, and 3B genes of a genetically uncharacterized EV-A71 isolate were designed using the Turbo si-Designer algorithm and synthesized by Bioneer (Daejeon, Republic of Korea) (Figure 1). The Turbo si-Designer algorithm incorporates parameters such as base composition, the number of repetitive bases in a row, thermodynamic instability, energy profiling, and base preference to optimize siRNA design [21,22,23,24]. In addition to the Turbo si-Designer algorithm, we further refined siRNA selection based on established criteria described by Ui-Tei, Reynolds, and Amarzguioui. Specifically, we selected siRNAs with a 30–52% GC content and low internal stability at the 5′ end of the antisense strand, and we avoided long GC stretches or internal repeats. We also prioritized sequences with A or U at the 5′ end of the antisense strand and G or C at the 5′ end of the sense strand to ensure asymmetrical thermodynamic stability. Furthermore, positional nucleotide preferences (such as A-/U-rich regions near the 5′ end of the antisense strand and absence of G at critical positions) were considered. Seed regions (positions 2–8) were checked for off-target potential via BLAST against the human transcriptome to ensure high specificity.

Each siRNA candidate targeting EV-A71 underwent a BLAST search by NCBI to ensure specificity and avoid off-target effects. The selected siRNA sequences were verified through BLAST searches to confirm that they did not target the human genome. Homology analysis was performed against all other sequences in the genome using a nonredundant sequence database. The nonsilencing scrambled siRNA (scr siRNA) sequence was designed to have no homology with the EV-A71 genome sequence available at NCBI and to have the same base composition as VP4-132 siRNA, one of the siRNAs designed in this study. All siRNA sequences were designed with 3′-dTdT overhang extensions against the 3′ UTR and a fluorescein-labeled FAM (fluorescein amidite) at the 5′ UTR end of the sense strand. The siRNA was synthesized by Bioneer Company, Inc., Daejeon, Republic of Korea, reconstituted in ultrapure distilled water at a concentration of 100 µM, and stored at −80 °C until use.

**Table 1:** siRNA sequences and corresponding target gene positions in the enterovirus 71 genome.

siRNA Name	Target Nucleotide Sequence (5′-3′)	GC Ratio (%)	Target Gene	Genomic Position
scrambled control	AUUCUAUCACUAGCGUGAC	38.1	None	None
VP4-120	CUGGAAAGCAAAGUCUCA	40	VP4	865–882
VP4-132	GUCUCAAACAAGAUCCUGA	38.1	VP4	877–895
VP4-153	AGUUUGCGAACCCUGUGAA	42.86	VP4	898–916
VP3-224	CAGUUGUGUGGAUAUUACA	33.33	VP3	2019–2037
VP3-198	GUCCUUGGCAAUCCACCAU	47.62	VP3	1993–2011
VP3-233	GGAUAUUACACCCAAUGGU	38.1	VP3	2028–2046
2B-201	CAGCCACACUAGCUCUGAU	47.62	2B	3979–3997
2B-114	CUGUUGAGAAGAUCUUGAA	33.33	2B	3892–3910
2B-3	GGGUAUCUGAUUACAUCAA	33.34	2B	3781–3799
3A-150	CACCAACUAAUGUGGAACG	42.86	3A	5203–5221
3A-111	GACAGUACUGCAGGGAACA	47.62	3A	5164–5182
3A-25	CAGGCCUAUAAGAAUUAGU	33.34	3A	5078–5096
3B-34	GAAGCCCGUGUUAAGAACA	42.86	3B	5357–5375
3B-10	UGGAGCGCCCAAGCAAAUU	47.62	3B	5333–5351
3B-38	CCCGUGUUAAGAACAGCCA	47.62	3B	5361–5379

Note. Genomic positions correspond to those of the BrCr strain, GenBank accession No. AB204852.1 and GU434678.1.

### 2.4. Transfection of siRNA

For determination of the effect of the siRNAs on viral replication, HeLa cells transfected with siRNA were infected with EV-A71 or CVB3 in independent assays. Transfection of siRNAs prior to virus infection into HeLa cells was performed using Lipofectamine RNAiMAX 2000 reagents (Invitrogen Life Technologies) following the manufacturer’s instructions. After 24 h transfection, the HeLa cells were incubated at 37 °C until further analysis of viral replication inhibition. HeLa cells were infected with EV-A71 at a multiplicity of infection (MOI) of 1 × 10^−3^ and with CVB3 at an MOI of 1 × 10^−5^. As a negative control, mock-infected cells were included. Cells were harvested at 24, 36, and 48 h post-infection, and inhibition of viral replication was assessed using Western blot, immunofluorescence assay, and plaque assay techniques.

### 2.5. Flow Cytometry

HeLa cells were transfected with different concentrations (0, 5, 10, 25, 50, 100, 200, and 300 nM) of FAM-labeled scrambled control siRNAs. At 72 h after transfection, cells were trypsinized and harvested. We counted the number of FAM-positive cells using flow cytometry. Flow cytometry analysis was performed using the LSR II flow cytometry device (BD Biosciences, San Jose, CA, USA) at the core facility for cell to in vivo imaging.

### 2.6. Plaque Assay

A plaque assay was conducted to assess the efficacy of siRNA. The viral titers were determined by performing an agar overlay plaque assay on Vero cells (CCL-81™, ATCC) to measure viral infectivity. Briefly, the supernatant from EV-A71- or CVB3-infected cells was serially diluted 10-fold and overlaid onto a 90 to 95% confluent monolayer of Vero cells. After incubation for 1 h, the Vero cells were washed with PBS and then overlaid with a mixture of 2X DMEM (Welgene, Gyeongsan, Republic of Korea) and 2% agar (Lonza, Walkersville, MD, USA) in each well. The cells were further incubated at 37 °C for 72 h, fixed with a 3.75% formaldehyde solution (Sigma–Aldrich, St. Louis, MO, USA), and stained with 1% crystal violet (Lugen Sci, Seoul, Republic of Korea). Plaques were counted, and the viral titer was calculated as plaque-forming units per milliliter. The viral titers of culture supernatants obtained from infected cells were determined in triplicate.

### 2.7. Immunofluorescence

Cells were seeded on a chamber slide one day prior to siRNA transfection, with or without virus infection. After transfection and virus infection, the cells were washed twice with PBS and fixed with 4% paraformaldehyde for 10 min. The fixed cells were then permeabilized with 0.1% Triton X-100 (Sigma–Aldrich) in PBS with 0.05% Tween^®^ 20 (PBST, Sigma–Aldrich) for 10 min, followed by blocking with 1% bovine serum albumin (Sigma–Aldrich) in PBST for 30 min at 25 °C. Subsequently, the cells were incubated overnight at 4 °C with an EV-A71 antibody (ab36367, Abcam, Cambridge, UK). After washing, the cells were incubated with goat anti-mouse IgG (H+L) cross-adsorbed secondary antibody (Invitrogen Life Technologies) for 1 h at 25 °C. The nuclei were stained with Hoechst 33342 (Invitrogen Life Technologies) for 10 min at 25 °C. The cells were visualized using an epifluorescence microscope (Carl Zeiss, Jena, Germany) and analyzed using the Axio Vision software version 4.0 (Carl Zeiss).

### 2.8. Western Blot Analysis

Cells were lysed with M-PER reagent (Invitrogen Life Technologies) containing a protease/phosphatase inhibitor cocktail (Roche Diagnostics, Indianapolis, IN, USA) on ice for 20 min. Cell lysates were then harvested by scraping, followed by centrifugation at 14,000× *g* for 15 min at 4 °C. The protein concentration was determined using a BCA Protein Assay (Intron Biotechnology, Seongnam-si, Republic of Korea). Equal amounts of protein were subjected to sodium dodecyl sulfate–polyacrylamide gel electrophoresis and transferred to polyvinylidene difluoride membranes (Bio-Rad Laboratories). The membranes were blocked for 1 h with 0.05% Tween 20 (Bio-Rad Laboratories) in Tris-buffered saline (TBS, Bio-Rad Laboratories) containing 5% milk (Bio-Rad Laboratories). Subsequently, the blots were incubated overnight at 4 °C with primary antibodies, followed by a 1 h incubation with a secondary antibody. Immunoreactive bands were visualized using enhanced chemiluminescence (GE Healthcare, Madison, WI, USA). Western blotting was performed using the following primary antibodies: EV-A71-VP1 (Gene Tex, Irvine, CA, USA), CVB3-VP1 (Mediagnost, Reutlingen, Germany), protein kinase R (PKR, dsRNA-activated protein kinase, Santa Cruz Biotechnology, Santa Cruz, CA, USA), *p*-PKR (Abcam), and β-actin (Santa Cruz Biotechnology). The secondary antibodies used in this study were anti-rabbit IgG, HRP-linked antibody (Cell Signaling Technology, Danvers, MA, USA) and anti-mouse IgG, HRP-linked antibody (Cell Signaling Technology).

### 2.9. Evaluation of Cytopathic Effects and Cell Viability

The protection of HeLa cells against cytopathic effects by various siRNAs over a time course of EV-A71 infection was assessed through microscopic observation. In addition, cell viability was evaluated to determine the optimal siRNA concentration for antiviral assays and to assess the cytopathic effects of EV-A71-infected cells. Similarly, post-treatment cell viability was assessed to confirm that the reduction in viral titers resulted from the action of siRNAs rather than a decrease in the number of cells at the end of each assay. Cell viability was measured using an EZ-Cytox cell viability assay kit (Daeillab Service, Seoul, Republic of Korea) according to the manufacturer’s instructions.

### 2.10. Evaluation of Off-Target Effect (Activation of the Interferon Pathway)

For investigating whether the inhibition of EV-A71 by siRNA treatment was a nonspecific effect related to the interferon response, the phosphorylation pattern of PKR was examined as a dsRNA-activated protein kinase.

### 2.11. Statistical Analyses

Three independent assays were conducted in triplicate, and statistical analyses were performed using a standard statistical software package (Prism Version 6; GraphPad Software, San Jose, CA, USA). For determination of the significance of differences, Student’s *t* test and two-way ANOVA followed by Bonferroni’s post hoc test or one-way ANOVA with Bonferroni’s multiple comparison test were employed.

## 3. Results

### 3.1. Design of siRNAs Against EV-A71

Based on the sequencing of the novel EV-A71 strain, we designed siRNAs targeting five selected genes. Three target-specific siRNA candidates were designed for each of the five genes using Turbo si-Designer, an siRNA algorithm program (Figure 1 and Table 1). For the selection of siRNA candidates, we took into consideration the guanine–cytosine ratio (GC%) of the siRNA sequence, as it is known to be inversely proportional to the RNAi activity [25,26,27]. Thus, siRNA candidates with a GC content ranging from 30% to 50% were chosen. A nonsilencing scr siRNA was designed to evaluate specific inhibitory effects.

### 3.2. Optimization of siRNA Concentration to Minimize Cytotoxicity and Off-Target Effects

**Figure 2:**
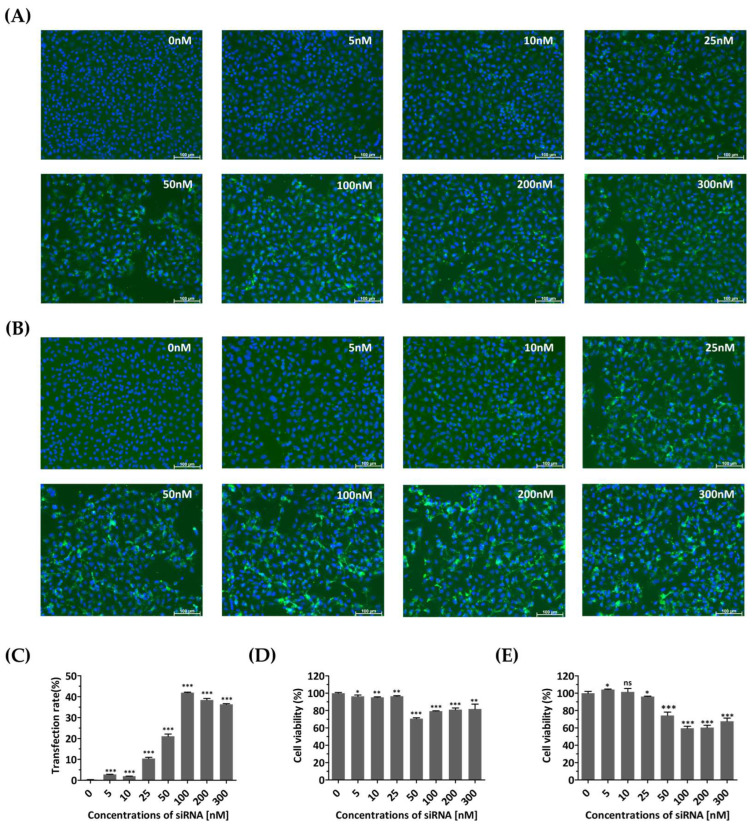
Monitoring of cells transfected with diverse concentrations of siRNA. HeLa cells were transiently transfected with different concentrations (0, 5, 10, 25, 50, 100, 200, and 300 nM) of FAM (fluorescein amidite)-labeled scrambled control siRNAs. The control group (0 nM) was not treated with siRNA and received only Lipofectamine. (**A**,**B**) Evaluation of siRNA effects using fluorescence detection of FAM. Confocal microscopy images showing green fluorescence indicating the expression of siRNA at 48 h (**A**) and 72 h post-transfection (**B**). The nucleus was stained with Hoechst 33342. Scale bar represents 100 μm. (**C**) The proportions of FAM-labeled HeLa cells transfected different concentrations of FAM-labeled scrambled control siRNAs at 72 h post-transfection were shown using flow cytometry. (**D**,**E**) Cytotoxicity assessment of siRNA-transfected HeLa cells. Cellular viability was quantified using a water-soluble tetrazolium salt assay at 48 h (**D**) and 72 h post-transfection (**E**). The results are presented as the percentage of viable cells compared to that of the siRNA-untreated group (0 nM) and are represented as the means ± SDs from three independent experiments. Statistical significance is indicated by asterisks, denoting *t* test *p* values (*, *p* < 0.05; **, *p* < 0.01; ***, *p* < 0.001; ns, not significant).

Cell growth and viability were evaluated to determine whether the transfection agent or siRNAs introduced into the cells caused any nonspecific cytotoxic effects. At 48 and 72 h post-transfection, FAM fluorescence showed a clear, dose-dependent increase up to 100 nM, compared to that of untreated cells (Figure 2A–C). Cells treated with siRNA concentrations above 50 nM showed relatively low viability (Figure 2D,E). In contrast, treatment with siRNA at a concentration of 25 nM resulted in high cell viability, with values of 96.45 ± 0.4662% and 96.13 ± 0.2942% after transient transfection for 48 and 72 h, respectively. Therefore, a concentration of 25 nM was determined as the optimal siRNA treatment concentration due to its minimal toxicity and sustained expression for up to 72 h.

### 3.3. siRNA Treatments Inhibit EV-A71 Replication and Decrease Viral Protein Expression

**Figure 3:**
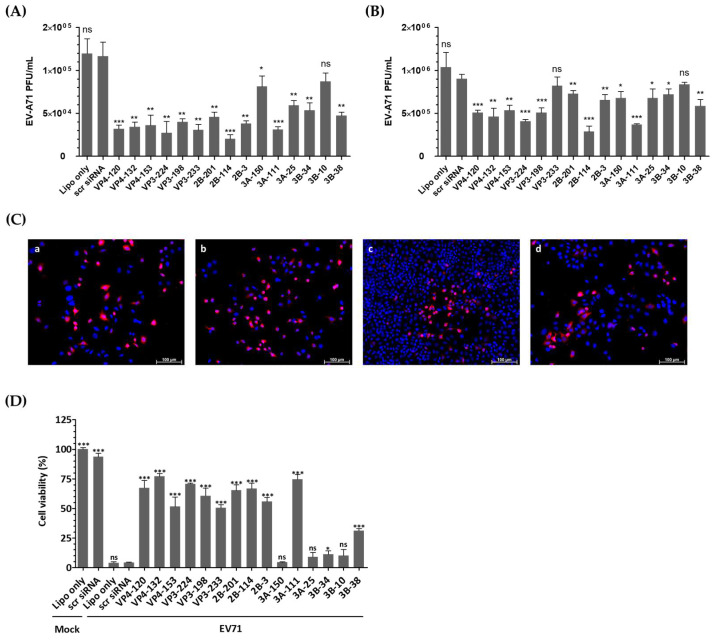
Evaluation of the antiviral activity of individual siRNAs against enterovirus A71 (EV-A71) infection. HeLa cells were transiently transfected with 25 nM siRNA for 24 h prior to virus infection. Subsequently, the cells were infected with EV-A71 at a multiplicity of infection of 0.001. (**A**,**B**) Extracellular viral titers were measured by plaque assays in the cell supernatants at 24 hpi (**A**) and 48 hpi (**B**). As controls, HeLa cells treated with Lipofectamine alone (Lipo only) and cells treated with scrambled siRNA (scr siRNA) alone were infected with the virus. The viral titers are presented as plaque-forming units per milliliter (PFU/mL), quantified from the supernatants of infected cells. The data represent the mean ± SD of three independent experiments. (**C**) Immunofluorescence analysis was performed at 36 hpi to visualize EV-A71 replication and siRNA-mediated inhibition. Cells were stained with EV-A71 monoclonal antibody (red) and Hoechst 33342 (blue) and visualized using a fluorescence microscope. The images show Lipofectamine-treated HeLa cells without siRNA (**a**), scr-siRNA-treated HeLa cells (**b**), 2B-114 siRNA-treated HeLa cells (**c**), and 3A-150 siRNA-treated HeLa cells (**d**). The scale bar represents 100 μm. (**D**) Cell survival at 48 h post-infection is presented as the percentage of viable cells relative to scr-siRNA-transfected controls for each siRNA-treated group. The mock-infected group represents cells without virus infection. Data are expressed as mean ± SD from three independent experiments. Statistical significance is indicated by asterisks, denoting *t* test *p* values (*, *p* < 0.05; **, *p* < 0.01; ***, *p* < 0.001; ns, not significant).

Figure 3A demonstrates that transfection of HeLa cells with EV-A71-specific siRNA resulted in a significant reduction in infectious progeny virus at 24 h post-infection (hpi). Consistently, as shown in Figure 3B, the viral titers obtained from the supernatants of the cells treated with VP4-132, VP3-224, 2B-114, and 3A-111 siRNAs were reduced to (4.63 ± 0.96) × 10^5^, (4.07 ± 0.20) × 10^5^, (2.87 ± 0.65) × 10^5^, and (3.70 ± 1.00) × 10^5^ PFU/mL, respectively, at 48 h post-infection, compared to (9.03 ± 0.05) × 10^5^ PFU/mL in HeLa cells treated with scr siRNA. In contrast, the cells transfected with VP3-233 and 3B-10 siRNA showed no inhibitory effect on EV-A71 replication. The most potent suppression of EV-A71 was observed in the cells treated with 2B-114 siRNA, followed by 3A-111, VP3-224, and VP4-132. At 36 h post-infection, immunofluorescence imaging was conducted to visualize viral replication before the onset of widespread cytopathic damage (Figure 3C). HeLa cells treated with 2B-114 siRNA exhibited markedly reduced VP1 fluorescence and higher cell density compared to the scr-siRNA-treated cells, indicating both effective viral inhibition and enhanced cell survival. It is important to note that all groups were seeded at the same initial density, and the observed differences in cell number are attributable to severe cytopathic effects in the Lipofectamine-treated HeLa cells without siRNA and scr-siRNA-treated groups, whereas siRNA-mediated inhibition effectively protected cells from virus-induced cytotoxicity.

### 3.4. siRNAs Delayed Cytopathic Effects and Increased the Viability of EV-A71-Infected HeLa Cells

The viability of HeLa cells was evaluated after transfection and infection to ensure that changes in viral titers were attributable to the effects of siRNA rather than cell integrity failures. After 48 h of infection, the HeLa cells untreated with siRNA or treated with scr siRNA showed over 95% cell death compared to the uninfected cells (Figure 3D). However, the cells pretreated with EV-A71-specific siRNA exhibited resistance to infection, as evidenced by high cell viability. Specifically, treatment with VP4-132, VP3-224, 2B-114, and 3A-111 siRNA increased cell viability to 77.05 ± 1.385%, 70.71 ± 0.3483%, 66.72 ± 2.635%, and 74.67 ± 2.234%, respectively.

### 3.5. No Activation of the Interferon Pathway When HeLa Cells Were Treated with siRNA

**Figure 4:**
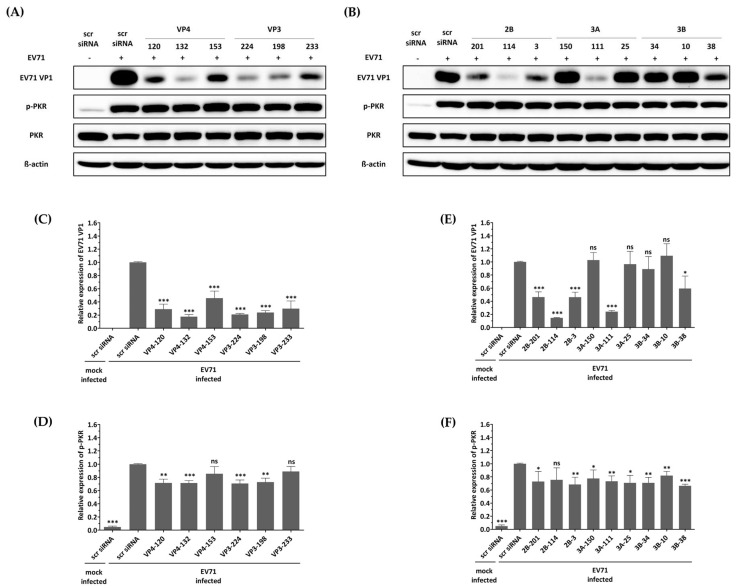
Effect of siRNAs on immune response and viral protein synthesis in enterovirus A71 (EV-A71)-infected HeLa cells. HeLa cells were transfected with each siRNA at a concentration of 25 nM. Twenty-four hours post-transfection, the cells were infected with EV-A71 at a multiplicity of infection of 0.001. Western blot analysis was performed on cell lysates collected at 24 h post-infection to assess the levels of PKR, phospho-PKR (p-PKR), and EV-A71 VP1. The relative protein quantities of the cells with siRNAs targeting structural proteins are shown in (**A**), while those with siRNAs targeting nonstructural proteins are shown in (**B**), compared to scrambled (scr)-siRNA-treated cell lysates. β-Actin was detected as the internal control. (**C**–**F**) The intensity of each band was quantified using the Image J software (version 1.53). The VP1 protein expression levels were normalized by β-actin (**C**,**E**). The p-PKR protein expression levels were normalized by PKR (**D**,**F**). All data are presented as means ± SD from three independent experiments, compared with the scr siRNA group. Statistical significance is indicated by asterisks, denoting *t* test *p* values (*, *p* < 0.05; **, *p* < 0.01; ***, *p* < 0.001; ns, not significant).

The first reported off-target effect of siRNA was the activation of the mammalian innate immune system, specifically the interferon response, by canonical siRNA duplexes [28,29,30]. Since the activation of interferon is known to act as an antiviral factor, we confirmed whether the inhibition of EV-A71 by siRNA treatment was a nonspecific effect according to the interferon response by discriminating the phosphorylation pattern of the PKR protein. As shown in Figure 4, no significant change in phosphorylated PKR protein was observed in cells transfected with different siRNAs exhibiting various EV-A71 inhibitory effects. These results indicate that each of the siRNAs did not lead to interferon response, and viral inhibition was exclusively mediated through siRNA. On the other hand, it was confirmed that phosphorylation of PKR protein was significantly increased in EV-A71-infected cells compared to uninfected cells, and native PKR protein was decreased in cells in which a relatively large amount of VP1 protein was recognized. CVB3 with a genome with partial low homology within the targeted regions can evade potent inhibitory effects.

**Figure 5:**
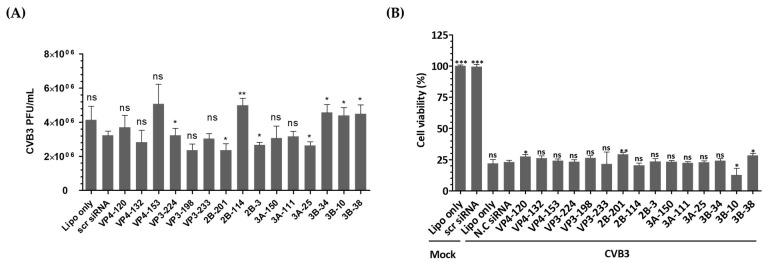

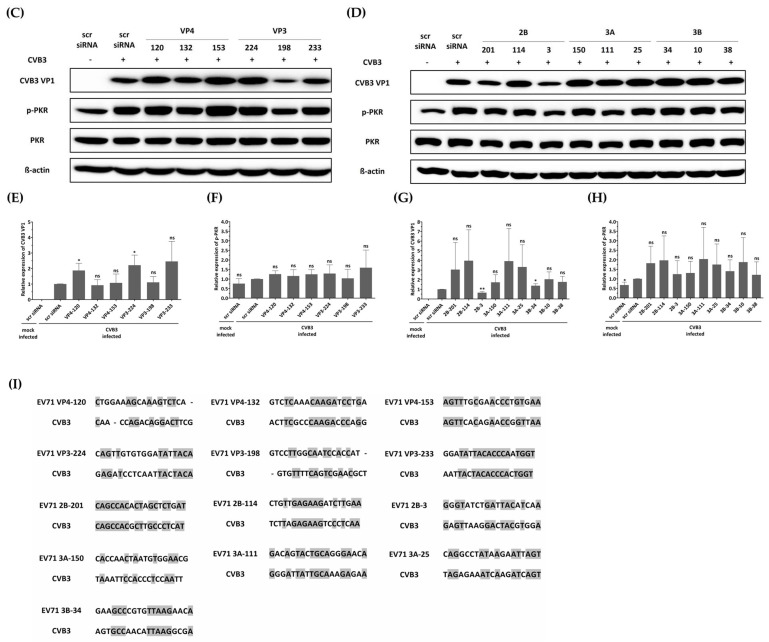
Evaluation of the antiviral activity of enterovirus A71 (EV-A71)-specific siRNAs against coxsackievirus B3 (CVB3) infection. HeLa cells were transiently transfected with 25 nM siRNA for 24 h prior to virus infection and then infected with CVB3 at a multiplicity of infection of 0.00001. (**A**) Cell supernatants were harvested at 48 hpi, and extracellular viral titers were measured by plaque assays. As controls, HeLa cells treated with Lipofectamine alone (Lipo only) and cells treated with scr siRNA alone were infected with the virus. The viral titers are presented as plaque-forming units per milliliter (PFU/mL), quantified from the supernatants of infected cells. The data represent the mean ± SD of three independent experiments. Statistical significance is indicated by asterisks, denoting *t* test *p* values (*, *p* < 0.05; **, *p* < 0.01; ***, *p* < 0.001; ns, not significant). (**B**) After 48 h of infection, cell survival was measured using a water-soluble tetrazolium salt assay. The mock-infected group represents cells without virus infection. The results are shown as the percentage of viable cells compared to scr-siRNA-transfected cells for each siRNA-transfected cell. Data represent the mean ± SD of three independent experiments. (**C**,**D**) Western blot analysis was performed on cell lysates collected at 24 hpi to assess the levels of PKR, phospho-PKR (p-PKR), and CVB3 VP1. The relative protein quantities of siRNA-treated cells targeting CVB3 structural proteins are shown in (**C**), while those targeting CVB3 nonstructural proteins are shown in (**D**), compared to scr-siRNA-treated cell lysates. β-Actin was detected as the internal control. (**E**–**H**) The intensity of each band was quantified using the Image J software (version 1.53). The VP1 protein expression levels were normalized by β-actin (**E**,**G**). The p-PKR protein expression levels were normalized by PKR (**F**,**H**). All data are presented as means ± SD from three independent experiments, compared to scr-siRNA-treated cell lysates. Statistical significance is indicated by asterisks, denoting *t* test *p* values (*, *p* < 0.05; **, *p* < 0.01; ns, not significant). (**I**) Sequence information for CVB3 aligned with the EV-A71 target sequence. Residues that are conserved across sequences are highlighted in gray.

In this study, we investigated the inhibitory potential of siRNAs targeting genes in the EV-A71 genome and compared it to that of CVB3, another important member of the *Enterovirus* genus. To evaluate the sequence specificity and antiviral effects of siRNAs, we used a similar experimental approach for both EV-A71 and CVB3 infections. Our findings showed that CVB3 with a genome with partial low homology within the targeted regions could evade the potent inhibitory effects of single siRNAs. In contrast to EV-A71, CVB3 efficiently replicated in cells transfected with EV-A71-specific siRNAs (Figure 5A), leading to a significant reduction in cell viability (Figure 5B). Furthermore, there were no significant differences observed in the levels of the VP1 protein of CVB3 or the phosphorylation pattern of the PKR protein (Figure 5C–H). Analysis of the siRNA target sequence revealed mismatches at several locations, which likely contributed to the reduced antiviral efficacy against CVB3 (Figure 5I). These results indicate that the inhibitory effects of siRNAs are specific to their target sequences and that mismatches in the target region can lead to evasion of siRNA-mediated inhibition by the virus.

## 4. Discussion

siRNA technology can target various types of viral genomes, making it a versatile mechanism suitable for broad-spectrum antiviral therapy [31,32]. Moreover, siRNAs target shorter nucleic acid sequences rather than larger functional domains of viral proteins, potentially resulting in numerous targets even in small viral genomes [33,34]. Therefore, siRNA design is relatively straightforward compared to drugs targeting proteins, enabling prompt adaptation to mutations by merely altering the target sequence.

The siRNA design in this study targeted the VP4, VP3, 2B, 3A, and 3B genes, which are critical for EV-A71 replication but have not been extensively studied. The results of the present study demonstrated a significant inhibitory effect against EV-A71 genotype A infection in HeLa cells for all genes targeted by at least one of the three siRNAs, with accurate sequence matching (Figure 3 and Figure 4). Specifically, VP4-132, VP3-224, 2B-114, and 3A-111 consistently demonstrated antiviral effects when considering all assessments. Notably, the antiviral effects of siRNAs targeting different positions within the same viral protein were found to be dissimilar. This finding highlights the importance of increased accessibility to the target, such as perfect sequence matching or target viral genome structures, rather than solely focusing on the function of the target viral protein, and this is consistent with prior studies [24,34,35,36,37].

The 3B gene consists of 20 to 22 amino acids and encodes a VPg protein that is covalently linked to the 5′ end of the RNA genome [38]. In the replication of picornaviruses, including polioviruses, the VPg protein is essential for viral RNA replication, and it is known that mutagenesis of the sequence encoding VPg can reduce or eliminate infectious virus production [39,40,41]. However, in this experiment, it is presumed that the length of the siRNA target site, which could be designed, was unfortunately the shortest compared to other gene sequences, which was a limitation for efficient siRNA design. Therefore, further structural studies of the RNA encoding 3B, which is yet to be elucidated, are required to resolve this.

In order to test the sequence-specificity of siRNAs for their antiviral effects, we evaluated CVB3, which belongs to the human enterovirus B lineage, in the same way as the EV-A71 infection experiments. As a result, CVB3 was shown to be fully replicable in EV-A71 specific siRNA transfected cells (Figure 5A) and significantly lowered the survival of CVB3-infected cells as measured by WST analysis (Figure 5B). Furthermore, there were no significant differences in the VP1 protein level of CVB3 and the PKR protein phosphorylation pattern (Figure 5C–I). An analysis of the siRNA target sequence indicates that there were mismatches at a number of locations that had implications on the antiviral efficacy (Figure 5E). This result confirmed that the inhibitory effect on EV-A71 was elicited through a sequence-specific reaction of siRNA, without ambiguous nonspecific side effects.

The cytopathic effect induced by EV-A71 was delayed by up to 48 h in HeLa cells transfected with the VP4-132, VP3-224, 2B-114, and 3A-111 siRNAs. This loss of protection likely results from siRNA dilution during cell division, as previously demonstrated through mathematical modeling of siRNA silencing kinetics in proliferating cells [42].

One of the main research objectives in the development of siRNA therapeutics is to minimize off-target effects [43]. As shown in Figure 2, we achieved this by using the lowest possible siRNA concentration while considering cell viability. Additionally, it is possible that the inhibition of viral replication is caused by an interferon-mediated response, which can be triggered by short dsRNA [44,45]. It is crucial to determine whether the siRNAs used in this study are capable of inducing off-target effects, such as activating the interferon response. Therefore, this study examined the phosphorylation of PKR to determine whether a nonspecific interferon response was induced. In the present study, neither an unintended interferon response nor inactivation of genes with low sequence homology occurred when siRNAs designed to target EV-A71 were employed.

In contrast, it was observed that phosphorylation of the PKR protein was significantly increased in EV71-infected cells compared to uninfected cells, and native PKR protein levels were decreased in cells where relatively high levels of VP1 protein were detected. These observations are consistent with recent research indicating that EV71 infection not only leads to PKR phosphorylation but also results in degradation of native PKR protein [46,47].

The lack of effective in vivo inhibition of virus proliferation by siRNAs remains a major limitation of this study. The use of siRNA-based anti-EV-A71 treatments in vivo is still under investigation. However, the abundance of antiviral studies utilizing siRNAs built upon these findings can enhance the feasibility of designing effective siRNAs with high efficiency and specificity, particularly in unexpected pandemic situations or when genetically uncharacterized variant strains emerge. To address this limitation, we plan to build on the present findings through future in vivo studies evaluating combination siRNA therapies targeting multiple independent viral regions.

The findings highlight the benefits of RNAi mechanisms in the development of treatments targeting viral diseases, providing versatility and numerous targets even in small viral genomes. This approach offers promise for future antiviral treatments in an unexpected infectious disease outbreak caused by an emerging virus or genetically uncharacterized variant strains. Moreover, given the sequence conservation among various enteroviruses, these results suggest that similar RNAi-based strategies may be applicable to other members of the *Enterovirus* genus, thereby broadening the therapeutic potential of siRNA beyond EV-A71.

## 5. Conclusions

In this study, we demonstrated the effective inhibition of EV-A71 replication in vitro using siRNAs targeting multiple viral genes, including VP4, VP3, 2B, and 3A. Among these, several siRNAs showed consistent antiviral activity, and their efficacy was closely associated with perfect sequence matching and target accessibility, rather than solely the functional role of the encoded proteins. Importantly, our results confirmed the sequence-specific nature of the siRNA-induced antiviral effect, without triggering off-target responses such as interferon pathway activation.

Furthermore, we observed that while the antiviral effects of individual siRNAs were transient due to dilution effects during cell division, their initial potency was significant. These findings underscore the potential of siRNA-based strategies as broad-spectrum antiviral therapeutics, especially for rapidly evolving RNA viruses like EV-A71. However, the limited in vivo applicability remains a challenge, necessitating further research on delivery methods and stability. Altogether, our findings provide foundational insights into siRNA design and its utility in developing targeted antiviral therapies in response to emerging infectious threats.

## Data Availability

Data is contained within the article and supplementary material. Further inquiries can be directed to the corresponding author.

## Supplementary Materials

The following supporting information can be downloaded at: https://www.mdpi.com/article/10.3390/biomedicines13071760/s1, Table S1: Genotype information that best matches the sequence registered in the GenBank database; Table S2: Primer sequences for PCR amplification and sequencing.

## Abbreviations

BLAST: Basic Local Alignment Search Tool; CVB3, coxsackievirus B3; DMEM, Dulbecco’s modified Eagle’s medium; EV-A71, enterovirus A71; FAM, fluorescein amidite; FBS, fetal bovine serum; GC%, guanine-cytosine ratio; hpi, hours post-infection; MOI, a multiplicity of infection; PKR, protein kinase R; RNAi, RNA interference; scr siRNA, scrambled siRNA; siRNA, small interfering RNA; TBS, Tris-buffered saline; UTR, untranslated regions.

## References

[1] McMinn P.C. (2002). An overview of the evolution of enterovirus 71 and its clinical and public health significance. FEMS Microbiol. Rev..

[2] Solomon T., Lewthwaite P., Perera D., Cardosa M.J., McMinn P., Ooi M.H. (2010). Virology, epidemiology, pathogenesis, and control of enterovirus 71. Lancet Infect. Dis..

[3] Wang S.M., Ho T.S., Lin H.C., Lei H.Y., Wang J.R., Liu C.C. (2012). Reemerging of enterovirus 71 in Taiwan: The age impact on disease severity. Eur. J. Clin. Microbiol. Infect. Dis..

[4] Chiu M.L., Luo S.T., Chen Y.Y., Chung W.Y., Duong V., Dussart P., Chan Y.F., Perera D., Ooi M.H., Thao N.T.T. (2020). Establishment of Asia-Pacific Network for Enterovirus Surveillance. Vaccine.

[5] Puenpa J., Wanlapakorn N., Vongpunsawad S., Poovorawan Y. (2019). The History of Enterovirus A71 Outbreaks and Molecular Epidemiology in the Asia-Pacific Region. J. Biomed. Sci..

[6] Geisbert T.W., Lee A.C., Robbins M., Geisbert J.B., Honko A.N., Sood V., Johnson J.C., de Jong S., Tavakoli I., Judge A. (2010). Postexposure protection of non-human primates against a lethal Ebola virus challenge with RNA interference: A proof-of-concept study. Lancet.

[7] Choi J.H., Croyle M.A. (2013). Emerging targets and novel approaches to Ebola virus prophylaxis and treatment. BioDrugs.

[8] Deng Y., Wang C.C., Choy K.W., Du Q., Chen J., Wang Q., Li L., Chung T.K., Tang T. (2014). Therapeutic potentials of gene silencing by RNA interference: Principles, challenges, and new strategies. Gene.

[9] Gish R.G., Satishchandran C., Young M., Pachuk C. (2011). RNA interference and its potential applications to chronic HBV treatment: Results of a Phase I safety and tolerability study. Antivir. Ther..

[10] Gebert L.F., Rebhan M.A., Crivelli S.E., Denzler R., Stoffel M., Hall J. (2014). Miravirsen (SPC3649) can inhibit the biogenesis of miR-122. Nucleic Acids Res..

[11] Gottlieb J., Zamora M.R., Hodges T., Musk A.W., Sommerwerk U., Dilling D., Arcasoy S., DeVincenzo J., Karsten V., Shah S. (2016). ALN-RSV01 for prevention of bronchiolitis obliterans syndrome after respiratory syncytial virus infection in lung transplant recipients. J. Heart Lung Transpl..

[12] DeVincenzo J., Lambkin-Williams R., Wilkinson T., Cehelsky J., Nochur S., Walsh E., Meyers R., Gollob J., Vaishnaw A. (2010). A randomized, double-blind, placebo-controlled study of an RNAi-based therapy directed against respiratory syncytial virus. Proc. Natl Acad. Sci. USA.

[13] Lu W.W., Hsu Y.Y., Yang J.Y., Kung S.H. (2004). Selective inhibition of enterovirus 71 replication by short hairpin RNAs. Biochem. Biophys. Res. Commun..

[14] Sim A.C., Luhur A., Tan T.M., Chow V.T., Poh C.L. (2005). RNA interference against enterovirus 71 infection. Virology.

[15] Tan E.L., Tan T.M., Chow V.T., Poh C.L. (2007). Enhanced potency and efficacy of 29-mer shRNAs in inhibition of Enterovirus 71. Antivir. Res..

[16] Wu Z., Yang F., Zhao R., Zhao L., Guo D., Jin Q. (2009). Identification of small interfering RNAs which inhibit the replication of several Enterovirus 71 strains in China. J. Virol. Methods.

[17] Deng J.X., Nie X.J., Lei Y.F., Ma C.F., Xu D.L., Li B., Xu Z.K., Zhang G.C. (2012). The highly conserved 5’ untranslated region as an effective target towards the inhibition of Enterovirus 71 replication by unmodified and appropriate 2’-modified siRNAs. J. Biomed. Sci..

[18] Liu H., Qin Y., Kong Z., Shao Q., Su Z., Wang S., Chen J. (2016). siRNA Targeting the 2Apro Genomic Region Prevents Enterovirus 71 Replication In Vitro. PLoS ONE.

[19] Tan E.L., Tan T.M., Tak Kwong Chow V., Poh C.L. (2007). Inhibition of enterovirus 71 in virus-infected mice by RNA interference. Mol. Ther..

[20] Zoll G.J., Melchers W.J., Kopecka H., Jambroes G., van der Poel H.J., Galama J.M. (1992). General primer-mediated polymerase chain reaction for detection of enteroviruses: Application for diagnostic routine and persistent infections. J. Clin. Microbiol..

[21] Elbashir S.M., Harborth J., Lendeckel W., Yalcin A., Weber K., Tuschl T. (2001). Duplexes of 21-nucleotide RNAs mediate RNA interference in cultured mammalian cells. Nature.

[22] Elbashir S.M., Lendeckel W., Tuschl T. (2001). RNA interference is mediated by 21- and 22-nucleotide RNAs. Genes Dev..

[23] Kurreck J. (2006). siRNA efficiency: Structure or sequence-that is the question. J. Biomed. Biotechnol..

[24] Wang X., Wang X., Varma R.K., Beauchamp L., Magdaleno S., Sendera T.J. (2009). Selection of hyperfunctional siRNAs with improved potency and specificity. Nucleic Acids Res..

[25] Amarzguioui M., Prydz H. (2004). An algorithm for selection of functional siRNA sequences. Biochem. Biophys. Res. Commun..

[26] Reynolds A., Leake D., Boese Q., Scaringe S., Marshall W.S., Khvorova A. (2004). Rational siRNA design for RNA interference. Nat. Biotechnol..

[27] Chan C.Y., Carmack C.S., Long D.D., Maliyekkel A., Shao Y., Roninson I.B., Ding Y. (2009). A structural interpretation of the effect of GC-content on efficiency of RNA interference. BMC Bioinform..

[28] Rao D.D., Senzer N., Cleary M.A., Nemunaitis J. (2009). Comparative assessment of siRNA and shRNA off target effects: What is slowing clinical development. Cancer Gene Ther..

[29] Reynolds A., Anderson E.M., Vermeulen A., Fedorov Y., Robinson K., Leake D., Karpilow J., Marshall W.S., Khvorova A. (2006). Induction of the interferon response by siRNA is cell type- and duplex length-dependent. RNA.

[30] Parashar D., Paingankar M.S., Kumar S., Gokhale M.D., Sudeep A.B., Shinde S.B., Arankalle V.A. (2013). Administration of E2 and NS1 siRNAs inhibit chikungunya virus replication in vitro and protects mice infected with the virus. PLoS Negl. Trop. Dis..

[31] Kang H., Ga Y.J., Kim S.H., Cho Y.H., Kim J.W., Kim C., Yeh J.Y. (2023). Small interfering RNA (siRNA)-based therapeutic applications against viruses: Principles, potential, and challenges. J. Biomed. Sci..

[32] Chatterjee K., Lakdawala S., Quadir S.S., Puri D., Mishra D.K., Joshi G., Sharma S., Choudhary D. (2023). siRNA-Based Novel Therapeutic Strategies to Improve Effectiveness of Antivirals: An Insight. AAPS PharmSciTech..

[33] Leonard J.N., Schaffer D.V. (2006). Antiviral RNAi therapy: Emerging approaches for hitting a moving target. Gene Ther..

[34] Qureshi A., Tantray V.G., Kirmani A.R., Ahangar A.G. (2018). A review on current status of antiviral siRNA. A review on current status of antiviral siRNA. Rev. Med. Virol..

[35] Ahmed F., Raghava G.P. (2011). Designing of highly effective complementary and mismatch siRNAs for silencing a gene. PLoS ONE.

[36] Lee H.S., Ahn J., Jee Y., Seo I.S., Jeon E.J., Jeon E.S., Joo C.H., Kim Y.K., Lee H. (2007). Universal and mutation-resistant anti-enteroviral activity: Potency of small interfering RNA complementary to the conserved cis-acting replication element within the enterovirus coding region. J. Gen. Virol..

[37] Rothe D., Wade E.J., Kurreck J. (2011). Design of small interfering RNAs for antiviral applications. Methods Mol. Biol..

[38] Chen C., Wang Y., Shan C., Sun Y., Xu P., Zhou H., Yang C., Shi P.Y., Rao Z., Zhang B. (2013). Crystal structure of enterovirus 71 RNA-dependent RNA polymerase complexed with its protein primer VPg: Implication for a trans mechanism of VPg uridylylation. J. Virol..

[39] Sun Y., Wang Y., Shan C., Chen C., Xu P., Song M., Zhou H., Yang C., Xu W., Shi P.Y. (2012). Enterovirus 71 VPg uridylation uses a two-molecular mechanism of 3D polymerase. J. Virol..

[40] Paul A.V., Peters J., Mugavero J., Yin J., van Boom J.H., Wimmer E. (2003). Biochemical and genetic studies of the VPg uridylylation reaction catalyzed by the RNA polymerase of poliovirus. J. Virol..

[41] Liu Y., Franco D., Paul A.V., Wimmer E. (2007). Tyrosine 3 of poliovirus terminal peptide VPg(3B) has an essential function in RNA replication in the context of its precursor protein, 3AB. J. Virol..

[42] Bartlett D.W., Davis M.E. (2006). Insights into the kinetics of siRNA-mediated gene silencing from live-cell and live-animal bioluminescent imaging. Nucleic Acids Res..

[43] Bartoszewski R., Sikorski A.F. (2019). Editorial focus: Understanding off-target effects as the key to successful RNAi therapy. Cell Mol. Biol. Lett.

[44] Sledz C.A., Holko M., de Veer M.J., Silverman R.H., Williams B.R. (2003). Activation of the interferon system by short-interfering RNAs. Nat. Cell Biol..

[45] Yang E., Li M.M.H. (2020). All About the RNA: Interferon-Stimulated Genes That Interfere with Viral RNA Processes. Front. Immunol..

[46] Chang Y.H., Lau K.S., Kuo R.L., Horng J.T. (2017). dsRNA Binding Domain of PKR Is Proteolytically Released by Enterovirus A71 to Facilitate Viral Replication. Front. Cell. Infect. Microbiol..

[47] Zhao X., Hu Y., Zhao J., Liu Y., Ma X., Chen H., Xing Y. (2024). Role of protein Post-translational modifications in enterovirus infection. Front. Microbiol..

