# Supplementary material for: Small Interfering RNAs Targeting VP4, VP3, 2B, or 3A Coding Regions of Enterovirus A71 Inhibit Viral Replication In Vitro
Source: Biomedicines. 2025 Jul 18;13(7):1760. doi: 10.3390/biomedicines13071760 (PMC12292190; doi:10.3390/biomedicines13071760)
Supplement: Supplementary file 1 [file biomedicines-13-01760-s001.zip › biomedicines-3638969-supplementary.pdf]

SUPPLEMENTARY MATERIALS

Small Interfering RNAs Targeting VP4, VP3, 2B, or 3A Coding Regions of Enterovirus A71 Inhibit Viral Replication In Vitro

Yun Ji Ga, Yun Young Go, and Jung-Yong Yeh

Supplementary Tables

Table S1. Genotype information that best matches the sequence registered in the GenBank database.

Gene Name	Description	GenBank ID	Genotype/Strain	Identities
5' UTR	Human enterovirus 71 genomic RNA, complete genome, sub_strain:BrCr-TR	AB204852.1	BrCr (BrCr-TR)	335/335 (100%)
	Human enterovirus 71 genomic RNA, complete genome, sub_strain:BrCr-ts	AB204853.1	BrCr (BrCr-ts)	334/335 (99%)
	Enterovirus A71 isolate EV71/wuhan/3018/2010, complete genome	KF501389.1	genotype: A	333/335 (99%)
	Human enterovirus 71 strain EV71-Hubei-09-China, complete genome	GU434678.1	EV71-Hubei-09-China	333/335 (99%)
	Human enterovirus 71 polyprotein gene, complete cds	U22521.1	BrCr	333/335 (99%)
VP4	Enterovirus A71 isolate EV71/wuhan/3018/2010, complete genome	KF501389.1	genotype: A	207/207 (100%)
	Human enterovirus 71 strain A-BrCr-USA-70 polyprotein gene, partial cds	JN874547.1	A-BrCr-USA-70 genotype: A	207/207 (100%)
	Human enterovirus 71 genomic RNA, complete genome, sub_strain:BrCr-TR	A B204852.1	BrCr (BrCr-TR)	207/207 (100%)
	Human enterovirus 71 polyprotein gene, complete cds	U22521.1	BrCr	207/207 (100%)
	Human enterovirus 71 strain EV71-Hubei-09-China, complete genome	GU434678.1	EV71-Hubei-09-China	203/207 (98%)
VP3	Enterovirus A71 gene for polyprotein, VP4, VP2, VP3, VP1 protein region, strain: BrCr	AB777928.1	BrCr	513/513 (100%)
	Human enterovirus 71 strain A-BrCr-USA-70 polyprotein gene, partial cds	JN874547.1	A-BrCr-USA-70 genotype: A	513/513 (100%)
	Human enterovirus 71 strain EV71-Hubei-09-China, complete genome	GU434678.1	EV71-Hubei-09-China	513/513 (100%)

	Human enterovirus 71 genomic RNA, complete genome, sub_strain:BrCr-ts	AB204853.1	BrCr (BrCr-ts)	513/513 (100%)
	Human enterovirus 71 genomic RNA, complete genome, sub_strain:BrCr-TR	AB204852.1	BrCr (BrCr-TR)	513/513 (100%)
	Human enterovirus 71 polyprotein gene, complete cds	U22521.1	BrCr	513/513(100%)
	Enterovirus A71 isolate EV71/wuhan/3018/2010, complete genome	KF501389.1	genotype: A	512/513 (99%)
2B	Enterovirus A71 isolate EV71/wuhan/3018/2010, complete genome	KF501389.1	genotype: A	300/300 (100%)
	Human enterovirus 71 strain EV71-Hubei-09-China, complete genome	GU434678.1	EV71-Hubei-09-China	300/300 (100%)
	Human enterovirus 71 genomic RNA, complete genome, sub_strain:BrCr-TR	AB204852.1	BrCr (BrCr-TR)	300/300 (100%)
3AB	Enterovirus A71 isolate EV71/wuhan/3018/2010, complete genome	KF501389.1	genotype: A	324/324 (100%)
	Human enterovirus 71 strain EV71-Hubei-09-China, complete genome	GU434678.1	EV71-Hubei-09-China	324/324 (100%)
	Human enterovirus 71 genomic RNA, complete genome, sub_strain:BrCr-TR	AB204852.1	BrCr (BrCr-TR)	323/324 (99%)

Table S2. Primer sequences for PCR amplification and sequencing

Primer Name	Direction	Sequence (5'-3')	Genomic Position*	Regions
EV_5'UTR_436	F	CAAGCACTTCTGTTTCCCCGG	167-187	5' UTR
EV_5'UTR_436	R	ATTGTCACCATAAGCAGCCA	583-602	5' UTR
VP4_439	F	GCCATCCAGTGTCAAACAGA	635-654	5' UTR
VP4_439	R	GTATGAAGGCCACTCACCATAC	1052-1073	VP2
VP3_736	F	GTTCTGAGTTTGCGGGTCT	1675-1693	VP2
VP3_736	R	CTGGTGTCCTTGCACAATTTC	2390-2410	VP1
2B_547	F	CTGGTGATTGTGGCGGTATT	3649-3668	2A
2B_547	R	GCAGCTGGAATGATCTTCTCTT	4174-4195	2C
3AB_524	F	TGGTGTGTGGTAAGGCTATTC	4927-4947	2C
3AB_524	R	GTCAGTTTGCGCTTGTCTAATG	5429-5450	3C

*According to the EV71 genome sequence in the GenBank database, accession no. U22521.1, AB204852.1, and KF501389.1
